# Multi-center clinical evaluation of the Panther Fusion SARS-CoV-2/Flu A/B/RSV assay in nasopharyngeal swab specimens from symptomatic individuals

**DOI:** 10.1128/jcm.00827-23

**Published:** 2023-10-30

**Authors:** Anjana Sasidharan, Rangaraj Selvarangan, Kennah Konrad, Matthew L. Faron, Salika M. Shakir, David Hillyard, Robert K. McCall, Ian H. McHardy, David C. Goldberg, James J. Dunn, Alexander L. Greninger, Christine Lansang, Rachel Bogh, Carmelle V. Remillard

**Affiliations:** 1 Children’s Mercy Hospital, Kansas City, Missouri, USA; 2 The Medical College of Wisconsin, Milwaukee, Wisconsin, USA; 3 ARUP Laboratories, Salt Lake City, Utah, USA; 4 Geneuity Clinical Research Services, Maryville, Tennessee, USA; 5 Scripps Health, San Diego, California, USA; 6 Acutis Diagnostics, Hicksville, New York, USA; 7 Texas Children’s Hospital, Houston, Texas, USA; 8 University of Washington, Seattle, Washington, USA; 9 Hologic, Inc., San Diego, California, USA; Boston Children's Hospital, Boston, Massachusetts, USA

**Keywords:** SARS-CoV-2, influenza, respiratory syncytial virus, multiplex assay, molecular respiratory assay, clinical agreement, Panther Fusion

## Abstract

The symptomology is overlapping for respiratory infections due to severe acute respiratory syndrome coronavirus-2 (SARS-CoV-2), influenza A/B viruses, and respiratory syncytial virus (RSV). Accurate detection is essential for proper medical management decisions. This study evaluated the clinical performance of the Panther Fusion SARS-CoV-2/Flu A/B/RSV assay in nasopharyngeal swab (NPS) specimens from individuals of all ages with signs and symptoms of respiratory infection consistent with COVID-19, influenza, or RSV. Retrospective known-positive and prospectively obtained residual NPS specimens were collected during two respiratory seasons in the USA. Clinical performance was established by comparing Panther Fusion SARS-CoV-2/Flu assay results to a three-molecular assay composite comparator interpretation for SARS-CoV-2 and to the FDA-cleared Panther Fusion Flu A/B/RSV assay results for all non-SARS-CoV-2 targets. A total of 1,900 prospective and 95 retrospective NPS specimens were included in the analyses. The overall prevalence in prospectively obtained specimens was 20.7% for SARS-CoV-2, 6.7% for influenza A, and 0.7% for RSV; all influenza B-positive specimens were retrospective specimens. The positive percent agreement of the Panther Fusion assay was 96.9% (378/390) for SARS-CoV-2, 98.0% (121/123) for influenza A virus, 95.2% (20/21) for influenza B virus, and 96.6% (57/59) for RSV. The negative percent agreement was ≥98.5% for all target viruses. Specimens with discordant Panther Fusion SARS/Flu/RSV assay results all had cycle threshold values of ≥32.4 (by comparator or by Panther Fusion SARS/Flu/RSV assay). Only five co-infections were detected in the study specimens. The Panther Fusion SARS-CoV-2/Flu/RSV assay provides highly sensitive and specific detection of SARS-CoV-2, influenza A virus, influenza B virus, and RSV in NPS specimens.

## INTRODUCTION

Respiratory infections due to influenza viruses (influenza A and B viruses) and respiratory syncytial virus (RSV) are common and cause significant morbidity and mortality globally (1 – 6). When the outbreak due to severe acute respiratory syndrome coronavirus-2 (SARS-CoV-2) began in December 2019, public health officials were faced with a new readily transmissible respiratory virus that, in addition to being highly infectious, could lead to significant complications in the elderly and individuals with serious health risks (7).

The overlapping symptomology of SARS-CoV-2, influenza A and B viruses, and RSV, as well as the potential for both concomitant infections due to contemporaneous circulation (8, 9), have rendered definitive clinical diagnosis based on clinical presentation alone virtually impossible. As such, multiplex molecular testing for all four of these respiratory viral targets at once provides an ideal solution to meet the need for optimal patient management, for implementation of proper infection control measures, and for informed respiratory surveillance by public health agencies.

The Panther Fusion SARS-CoV-2/Flu A/B/RSV (“Panther Fusion SARS/Flu/RSV”) assay received FDA clearance in May 2023. The assay can detect nucleic acids from SARS-CoV-2, influenza A, influenza B, and RSV viruses from a single specimen. The objective of the multi-center study described here was to establish the performance characteristics of the Panther Fusion SARS/Flu/RSV assay in nasopharyngeal swab (NPS) specimens collected from symptomatic individuals.

## MATERIALS AND METHODS

### Prospective and retrospective clinical specimens

De-identified residual NPS specimens in viral transport media (VTM) were prospectively collected between November 2020 and March 2021 (*n* = 893) and between January 2022 and March 2022 (*n* = 1,056) from five geographically distinct US clinical centers: Acutis Diagnostics (Hicksville, NY), Children’s Mercy Hospital (Kansas City, MO), Medical College of Wisconsin (Milwaukee, WI), Scripps Clinic (La Jolla, CA), and Texas Children’s Hospital (Houston, TX). All specimens originated from individuals of all ages with suspected respiratory viral infection consistent with COVID-19, influenza, or RSV whose NPS specimens were submitted for standard of care (SOC) testing.

Residual NPS specimens meeting the following inclusion criteria were enrolled: the specimen was residual VTM [Copan Universal Transport Medium (UTM) and BD Universal Viral Transport Medium (UVT)] after SOC testing for respiratory infection; the specimen was held at 2°C to 8°C for less than or equal to 2 days prior to enrollment; and at least 1.75 mL of specimens remained after SOC testing. Specimens stored in saline were not used in this study. Retrospective virus-positive NPS specimens in VTM collected between December 2019 and March 2020 (*n* = 102; 763 to 836 days old, mean and median 800 days) were obtained to supplement the clinical performance evaluation for influenza A virus, influenza B virus, and RSV. All eligible retrospective NPS specimens had a confirmed positive result from an FDA-cleared molecular assay for at least one of the non-SARS-CoV-2 viral targets. No retrospective specimens positive for SARS-CoV-2 were used in the study.

**Fig 1:**
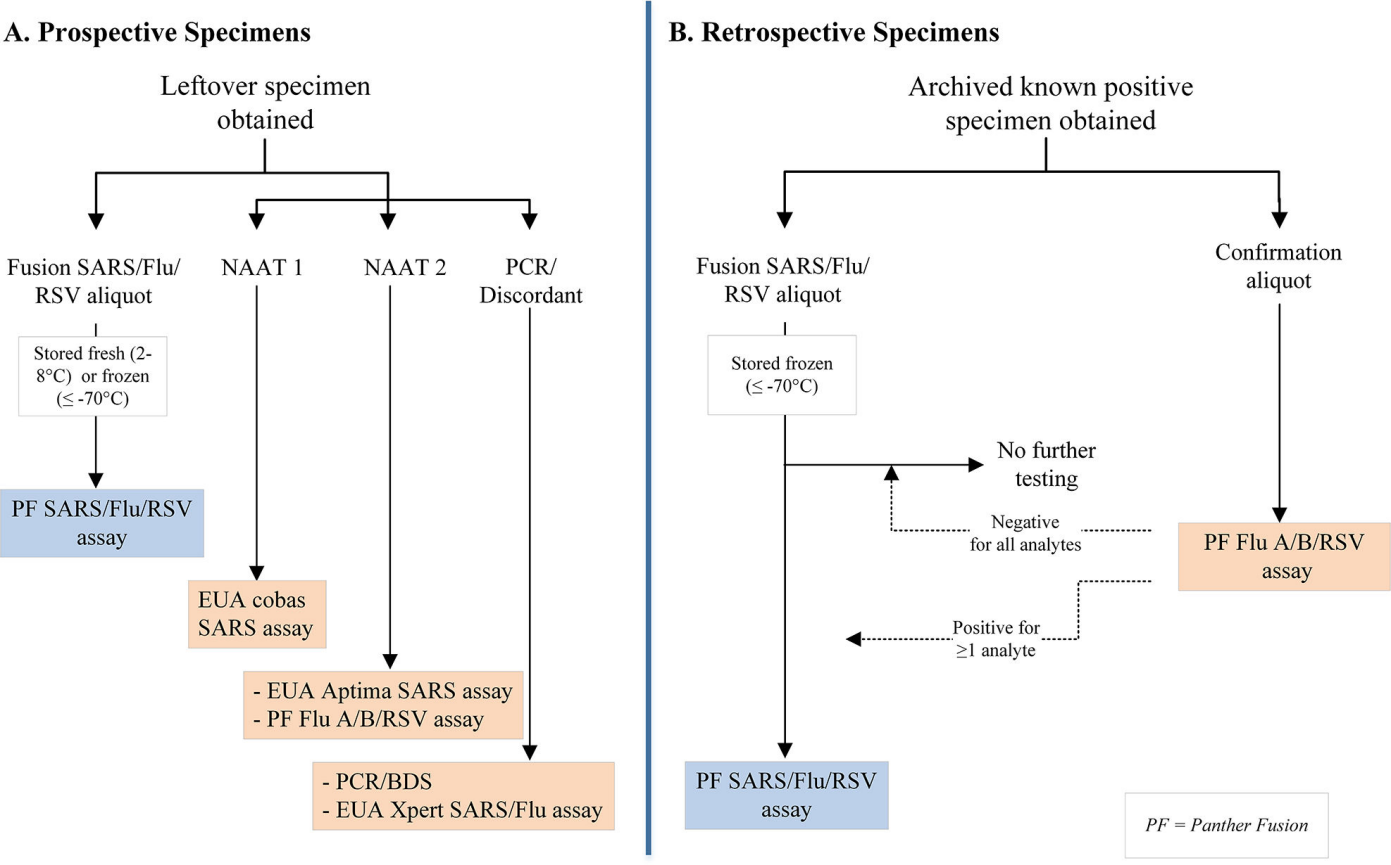
Specimen processing and testing workflow.

Demographic and limited clinical data were collected, including the date of specimen collection, age or age range at the time of collection, sex, the results [including cycle threshold (Ct) values if available] of SARS-CoV-2 testing performed as SOC, and the type of VTM. Specimens were processed and tested as shown in Fig. 1.

Prospective specimens were stored at 2°C to 8°C (42%; 4 to 9 days, median and mean 7 days) or at −70°C or colder (58%; 23 to 480 days, median 389 days, mean 335 days) prior to testing with the Panther Fusion SARS/Flu/RSV assay. All retrospective specimens were stored frozen at −70°C or colder for 761 to 837 days (median 800 days, mean 801 days) prior to testing.

### Panther Fusion SARS-CoV-2/Flu A/B/RSV testing

Each eligible NPS specimen was tested with the investigational Panther Fusion SARS/Flu/RSV assay at one of three US testing sites according to the assay’s instructions for use. The assay reported results for SARS-CoV-2 (targets: two regions within the ORF1ab gene, not differentiated), influenza A virus (target: matrix gene), influenza B virus (target: matrix gene), and RSV (target: matrix gene) detection separately.

### Comparator testing

With the exception of PCR/bidirectional sequencing, all comparator testing was completed at one central laboratory; PCR/bidirectional sequencing was performed at Hologic, Inc. All comparator assay testing and result interpretation were performed according to each assay’s software and/or manufacturer’s instructions for use. Study personnel performing the comparator assay testing were blinded to the Panther Fusion SARS/Flu/RSV assay results and all final comparator method results.

All prospective NPS specimens were tested with the FDA-designated emergency use authorization (EUA) cobas SARS-CoV-2 test (Roche Diagnostics), the Aptima SARS-CoV-2 assay (Hologic, Inc.), and the FDA-cleared Panther Fusion Flu A/B/RSV assay (Hologic, Inc.). For the SARS-CoV-2 target, specimens were also tested with a validated PCR/bidirectional sequencing assay if the cobas and Aptima assay results disagreed. Retrospective NPS specimens were tested with the Panther Fusion Flu A/B/RSV assay only; only retrospective specimens confirmed positive for at least one non-SARS-CoV-2 viral target were eligible for investigational Panther Fusion SARS/Flu/RSV assay testing (see Fig. 1B). Volume permitting, specimens with discordant results between the investigational assay and the comparator method for any viral target were tested with the FDA-designated EUA Xpert Xpress SARS-CoV-2/Flu/RSV assay (Cepheid).

### Result interpretation and discordant investigation

Because there is no gold standard method for the detection of SARS-CoV-2, a composite comparator method that included up to three valid results from the SARS-CoV-2 assays (cobas, Aptima, and PCR/bidirectional sequencing) was used to establish the SARS-CoV-2 infected status. If at least two of the three comparator SARS-CoV-2 assays were positive, the infected status was considered SARS-CoV-2 positive; if two comparator SARS-CoV-2 assay results were negative, the infected status was considered SARS-CoV-2 negative. Specimens without at least two valid concordant SARS-CoV-2 assay results were excluded from SARS-CoV-2 analyses. The infected status for influenza A virus, influenza B virus, and RSV was established using the Panther Fusion Flu A/B/RSV assay results.

Specimens with inconclusive comparator method results and those with invalid or missing investigational assay results were excluded from the analyses. A Panther Fusion SARS/Flu/RSV assay result was considered true positive (TP) or true negative (TN) only if it agreed with the comparator method result. A result was considered false positive (FP) or false negative (FN) when it disagreed with the comparator method result. Discordant investigation results and results from SOC testing were not used to recalculate performance data.

### Statistical analysis

Positive percent agreement (PPA) was calculated as 100 × TP/(TP + FN), while negative percent agreement (NPA) was calculated as 100 × TN/(TN + FP). Both PPA and NPA were calculated for SARS-CoV-2, influenza A virus, influenza B virus, and RSV separately. Binomial two-sided 95% confidence intervals (95% CIs) were calculated for PPA and NPA according to the score method (10). Prevalence was calculated as 100 × number of specimens with infection (by comparator method)/total number of specimens tested. Analyses were performed with SAS software (version 9.4; SAS Institute Inc., Cary, NC).

## RESULTS

### Specimen disposition

A total of 2,051 prospective and retrospective residual NPS specimens from unique individuals were obtained in this study. Forty-five specimens were withdrawn, primarily due to mishandling during transport to the testing sites. Non-withdrawn specimens with invalid Panther Fusion SARS/Flu/RSV assay results or an inconclusive infected status for a given viral target were considered non-evaluable and were excluded from the analyses. The most common reason for exclusion was an inconclusive, missing, or invalid comparator method result for a given viral target; only four specimens were excluded from all analyses because of final invalid Panther Fusion SARS/Flu/RSV assay results. Of the 2,006 non-withdrawn specimens, 1,900 prospective specimens and 95 retrospective specimens were evaluable for the performance analyses for SARS-CoV-2 (*n* = 1,887), influenza A virus (*n* = 1,865), influenza B virus (*n* = 1,858), and/or RSV (*n* = 1,883).

### Subject demographics

Table 1 provides summarized demographics and specimen information for all 1,995 evaluable prospectively collected and retrospective specimens. Just over half of the specimens were from female (55%, *n* = 1,100) subjects. Almost half of the subjects (43%, *n* = 851) were 21 years or younger, including 404 subjects below the age of 5 years; the mean subject age was 32.9 years (range 0 to 100 years). The majority (65%, *n* = 1,289) of the subjects had attended clinical centers in the US Midwest region.

**TABLE 1:** Demographics and specimen information^*b*^

Category	Overall	Prospective	Retrospective
Total, *N*	1,995	1,900	95
Age (years)			
Median (min–max)	30.0 (0–100)	29.0 (0–100)	48.0 (0–93)
Mean ± SD	32.9 ± 27.08	32.4 ± 26.94	43.0 ± 28.07
<5	404 (20.3%)	388 (20.4%)	16 (16.8%)
5–21	447 (22.4%)	435 (22.9%)	12 (12.6%)
22–40	387 (19.4%)	372 (19.6%)	15 (15.8%)
41–60	342 (17.1%)	326 (17.2%)	16 (16.8%)
≥61	415 (20.8%)	379 (19.9%)	36 (37.9%)
Sex			
Male	894 (44.8%)	850 (44.7%)	44 (46.3%)
Female	1,100 (55.1%)	1,049 (55.2%)	51 (53.7%)
Unknown	1 (0.1%)	1 (0.1%)	0
Geographic region			
Northeast (ADI)	181 (9.1%)	181 (9.5%)	0
Midwest (CMH, MCW)	1,289 (64.6%)	1,253 (65.9%)	36 (37.8%)
West (SCB)	503 (25.2%)	444 (23.4%)	59 (62.1%)
South (TCH)	22 (1.1%)	22 (1.1%)	0
Specimen age (days)			
Median (min–max)	47.0 (4–837)	40.0 (4–480)	800.0 (761–837)
Mean ± SD	225.7 ± 232.47	196.9 ± 198.32	801.1 ± 19.77
Specimen storage*^a^*			
Fresh	801 (40.2%)	801 (42.2%)	0
Frozen	1,194 (59.8%)	1,099 (57.8%)	95 (100%)


^*a*^ Refers to storage of specimens for Panther Fusion SARS/Flu/RSV assay testing only.

^*b*^ ADI, Acutis Diagnostics; CMH, Children’s Mercy Hospital; MCW, Medical College of Wisconsin; SCB, Scripps Clinic; TCH, Texas Children’s Hospital.

### Prevalence

The prevalence for each viral target, as established by the comparator method results, in prospective specimens varied by region (Table 2). The collection period for study specimens spanned two influenza seasons in the USA. The first collection period (November 2020 to March 2021) for SARS-CoV-2 occurred in the Midwest, West, and South regions with the following prevalence, respectively: 15.5%, 45.0%, and 4.5%. The second collection period (January 2022 to March 2022) for SARS-CoV-2 occurred in the Northeast and Midwest regions with the following prevalence, respectively: 6.6% and 13.6%. SARS-CoV-2 was prominent across both collection periods (30.1% prevalence in season 1 and 12.3% in season 2), whereas influenza A virus and RSV were only detected in the 2022 collection period; no influenza B virus-positive specimens were identified in either collection period. The total prevalence among prospectively collected specimens was 20.7% for SARS-CoV-2, 6.7% for influenza A virus, and 0.7% for RSV. Prevalence varied across age groups for each target virus (data not shown): influenza A virus prevalence was highest in those aged 5 to 21 (17.9%) years, RSV prevalence was highest in those aged less than 5 (1.3%) years and 5 to 21 (1.2%) years, and SARS-CoV-2 prevalence was highest in those aged 41 to 60 (30.5%) years and 61 years or more (33.7%).

**TABLE 2:** Study prevalence by region^*b*^

Geographic region^*a*^	Prevalence		
	SARS-CoV-2	Influenza A	RSV
Northeast (ADI)	11/173 (6.4%)	6/181 (3.3%)	6/181 (3.3%)
Midwest (CMH, MCW)	178/1,248 (14.3%)	117/1,216 (9.6%)	7/1,216 (0.6%)
West (SCB)	200/444 (45.0%)	0/418 (0.0%)	0/418 (0.0%)
South (TCH)	1/22 (4.5%)	0/22 (0.0%)	0/22 (0.0%)
Total	390/1,887 (20.7%)	123/1,837 (6.7%)	13/1,837 (0.7%)


^*a*^ Regions correspond to CDC surveillance regions: www.cdc.gov/surveillance/nrevss/rsv/region.html. Notes: Analysis includes prospective specimens only. Prevalence for influenza B is not shown as there were no influenza B-positive prospective specimens.

^*b*^ ADI, Acutis Diagnostics; CMH, Children’s Mercy Hospital; MCW, Medical College of Wisconsin; SCB, Scripps Clinic; TCH, Texas Children’s Hospital.

### Clinical performance

The clinical performance of the Panther Fusion SARS/Flu/RSV assay against the comparator methods is shown in Table 3 by viral target. Overall, the results from the Panther Fusion SARS/Flu/RSV assay were highly concordant with the comparator methods for all viral targets. The assay exhibited 96.9% (378/390) positive percent agreement (95% CI, 94.7% to 98.2%) and 98.5% (1,474/1,497) negative percent agreement (95% CI, 97.7% to 99.0%) against the composite comparator for SARS-CoV-2. The total positive percent agreement estimates against the FDA-cleared comparator were 98.0% (148/151; 95% CI, 94.3% to 99.3%) for influenza A virus, 95.2% (20/21; 95% CI, 77.3% to 99.2%) for influenza B virus, and 96.6% (57/59; 95% CI, 88.5% to 99.1%) for RSV; the negative percent agreement was greater than or equal to 98.5% for influenza A virus, influenza B virus, and RSV. The negative percent agreement varied minimally between specimens tested fresh or frozen for all viral targets: 98.3% vs 98.6% for SARS-CoV-2, 99.7% for both for influenza A virus, 99.7% vs 99.8% for influenza B virus, and 100% for both for RSV. The variation in positive percent agreement was also low between fresh and frozen specimens for SARS-CoV-2 (93.8% vs 97.5%) and influenza A virus (98.0% vs 100%); all RSV-positive prospective specimens were tested fresh, and no influenza B virus-positive prospective specimens were collected.

**TABLE 3:** Panther Fusion SARS/Flu/RSV assay performance for all viral targets^*b*^

Target virus	Specimen category	PPA		NPA	
		No. of true positive/total positive	% (95% CI)^*a*^	No. of true negative/total negative	% (95% CI)^*a*^
SARS-CoV-2	Prospective	378/390	96.9 (94.7, 98.2)	1,474/1,497	98.5 (97.7, 99.0)
Influenza A	Prospective	121/123	98.4 (94.3, 99.6)	1,709/1,714	99.7 (99.3, 99.9)
	Retrospective	27/28	96.4 (82.3, 99.4)	NA	NA
	Total	148/151	98.0 (94.3, 99.3)	NA	NA
Influenza B	Prospective	0/0	NC	1,833/1,837	99.8 (99.4, 99.9)
	Retrospective	20/21	95.2 (77.3, 99.2)	NA	NA
	Total	20/21	95.2 (77.3, 99.2)	NA	NA
RSV	Prospective	11/13	84.6 (57.8, 95.7)	1,824/1,824	100 (99.8, 100)
	Retrospective	46/46	100 (92.3, 100)	NA	NA
	Total	57/59	96.6 (88.5, 99.1)	NA	NA


^*a*^ Score CI.

^*b*^ Total, prospective and retrospective specimens combined; NA, not applicable; NPA, negative percent agreement; PPA, positive percent agreement.

**Fig 2:**
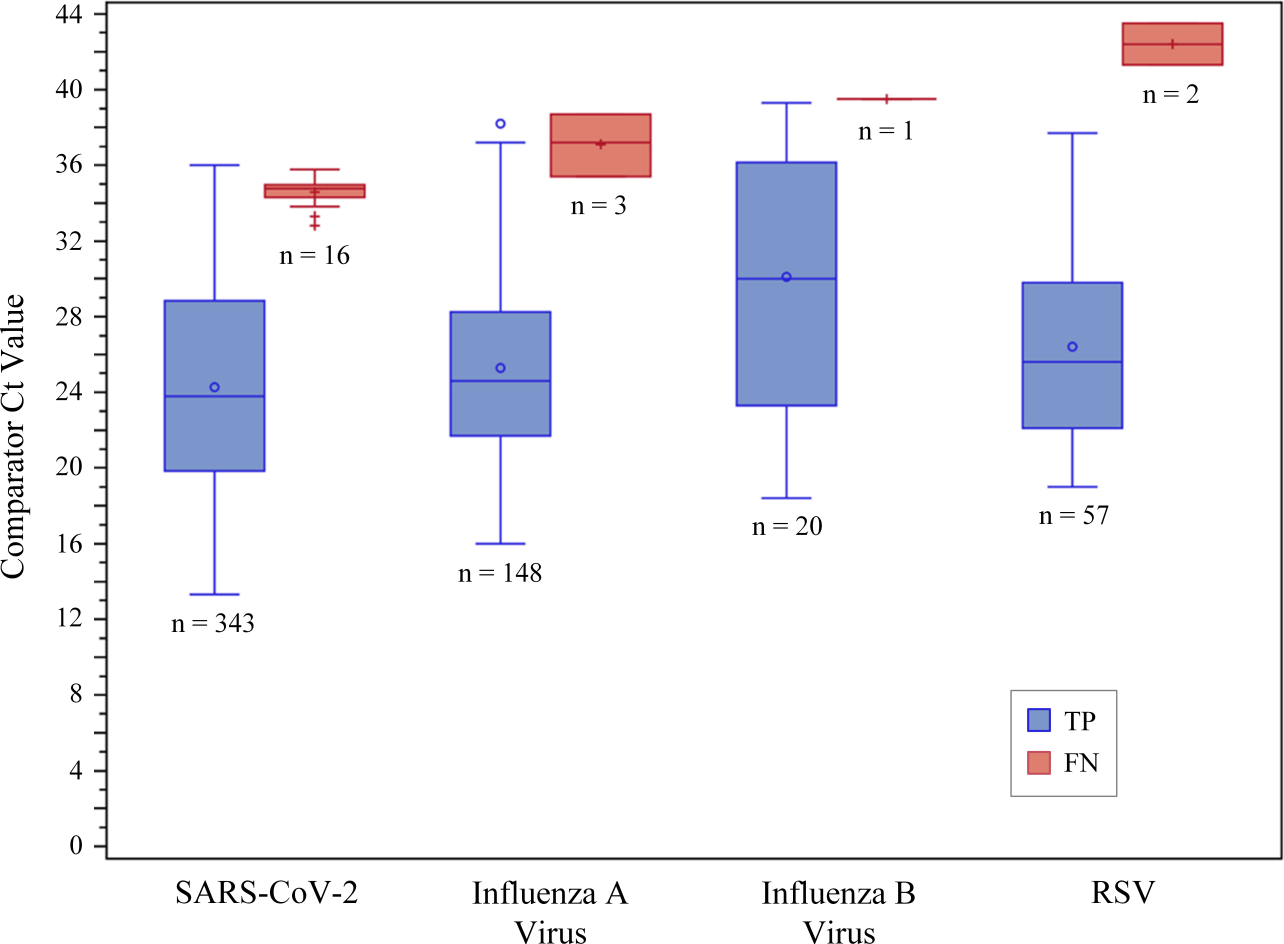
Distribution of comparator Ct values for true positive and false negative specimens. Box and whisker plot showing medians (middle line within each box), means (sign within each box), 25th and 75th percentiles (bottom and top of each box), and interquartile ranges ± 1.5 (whiskers) for each analyte and specimen group. Outliers are shown as symbols outside the interquartile ranges. Comparator Ct values were reported by the assay software for the EUA cobas SARS-CoV-2 (target 1: ORF1ab) for SARS-CoV-2 and the Panther Fusion Flu A/B/RSV assays for the influenza A virus, influenza B virus, and RSV targets. Of the three-assay comparator for SARS-CoV-2, only the cobas assay software reports Ct values; thus, the SARS-CoV-2 counts are based on 359 specimens with a valid positive result on the cobas SARS-CoV-2 assay and a matched valid result on the Panther Fusion SARS/Flu/RSV assay. Specimens with negative cobas SARS-CoV-2 assay target 1 results or presumptive positive results are excluded from this analysis.

Only 7 of 12 specimens with false negative results and 14 of 32 specimens with false positive results had sufficient remaining volume for discordant resolution testing with the EUA Xpert assay. All seven false negative specimens (five SARS-CoV-2, three influenza A virus, and one influenza B virus) were positive by this nucleic acid amplification test (NAAT), albeit with high Xpert Ct values: Ct 33 to 38.2 for SARS-CoV-2, Ct 38 for influenza A virus, and Ct 37.9 for influenza B virus. Although the false negative RSV specimens were not retested, their initial comparator Ct values were 41.3 and 43.5. Eight false positive SARS-CoV-2 specimens were positive by this NAAT (Ct values 36.3 to 44.2). All other false positive specimens tested (four SARS-CoV-2, two influenza A viruses, and one influenza B virus) were negative by the additional NAAT. Testing results for specimens with discordant results and Passing-Bablok regression Ct scatter plots (Panther Fusion SARS/Flu/RSV vs comparator) are listed in the supplemental material for each viral target. Fig. 2 shows the distribution of Ct values, shown as box and whisker plots for each target virus, reported by the comparator assays for specimens with true positive and false negative compared to the EUA cobas SARS-CoV-2 assay only for SARS-CoV-2 and to the Panther Fusion Flu A/B/RSV assay for all other targets.

### Co-infections detected by the Panther Fusion SARS/Flu/RSV assay

Five of the 1,900 evaluable prospective specimens tested were co-infected: four SARS-CoV-2^+^/influenza A virus^+^ specimens and one SARS-CoV-2^+^/influenza B virus^+^ specimen; three of the SARS-CoV-2^+^/influenza A virus^+^ co-infections were confirmed by comparator testing, one specimen was influenza A virus^+^, and one was SARS-CoV-2^+^. Two of the 95 evaluable retrospective specimens were co-infected: one influenza A virus^+^/influenza B virus^+^ and one influenza A virus^+^/RSV^+^; both co-infections were confirmed by comparator testing.

## DISCUSSION

Swift diagnosis and preventive measures are important in preventing the transmission of influenza virus, RSV, and SARS-CoV-2. Going forward, it is likely that SARS-CoV-2 will become an endemic disease, with the preponderance of transmission of SARS-CoV-2 likely to occur in winter months when it will co-circulate with influenza viruses and RSV (11, 12), with persistence of the viruses and the seasonality of the peaks fueled by pockets of susceptible individuals and waning immunity after infection or vaccination against current strains or phenotypes of the viruses. Because of the overlapping clinical presentations of SARS-CoV-2, influenza virus, and RSV infections, reliable detection and differentiation of these respiratory viruses using multiplex technologies is important for anticipated spikes in the circulation of these viruses during future epidemics. Prior to the COVID-19 pandemic, a number of cleared or approved highly sensitive and specific sample-to-answer assays and platforms for the detection and differentiation of influenza viruses A and B and RSV were available to testing laboratories. Since December 2019, 16 FDA-designated EUA multi-analyte *in vitro* diagnostic assays that detect SARS-CoV-2 and other respiratory viruses have been developed for clinical use (13). The BioFire Respiratory Panel 2.1 assay and the Panther Fusion SARS-CoV-2/Flu A/B/RSV assay are currently the only FDA-cleared assays that detect SARS-CoV-2 in addition to other respiratory infection viruses (14, 15).

Prospective collection for this multi-site study was conducted across two respiratory seasons, with only SARS-CoV-2 detected in both seasons. It should be noted that the first collection period (November 2020 to March 2021) was in the middle of the COVID-19 pandemic when SARS-CoV-2 circulation was very high and that the incidence of other respiratory viruses was nearly absent. The differences in prevalence observed between collection locations likely reflect local transmission patterns during the study periods and the efficacy of institutional diagnostic stewardship efforts.

In this comparative analysis, the Panther Fusion SARS/Flu/RSV assay exhibited a high level of concordance with the comparator method results for all viral targets, with PPA and NPA greater than or equal to 95.2% and 98.5%, respectively, for all the target viruses. There was minimal variation in the PPA and NPA between fresh and frozen specimens for all target viruses. This is noteworthy since frozen archived specimens are not recommended for studies aiming to determine clinical sensitivity or specificity because freeze-thawing can potentially alter the specimen’s characteristics and impact assay performance when compared to fresh specimens, the intended use for the test. Specimens that yielded discordant results for the three targets exhibited Ct values of ≥36.6 for SARS-CoV-2, ≥37.2 for influenza A virus, ≥39.5 for influenza B virus, and ≥42.4 for RSV with either the investigational or comparator assay. The high Ct values by the investigational and comparator assays for specimens with discordant results are suggestive of low viral loads for each target virus in these specimens. There were four co-infections noted, which were confirmed by comparator testing. The low number of co-infections of SARS-CoV-2 with either influenza or RSV is consistent with other reports in upper respiratory tract specimens (16 – 22).

There were limitations to note in this study. First, subtype information can be particularly valuable in an outbreak situation and in better describing the dominant circulating strains of the collection period in a study. No subtyping was completed for specimens positive for influenza A virus, influenza B virus, RSV, or SARS-CoV-2, so we can only surmise which variants were detected based on external surveillance data for the collection time. Second, although prospective enrollment occurred during two distinct respiratory seasons, both the decreased circulation of influenza and RSV during these periods may have contributed to the lower than “normal” number of influenza virus- and RSV-positive specimens and the small number of co-infections. Third, because the study utilized specimens leftover from standard of care testing, data on days since symptom onset, COVID-19 and influenza vaccination status, and reasons for standard of care testing (i.e., high-risk employment and contact case) were not available. Having these data may have provided insight, especially for the specimens with discordant results; for example, it may have clarified whether the discordant specimens with high Ct values were from someone with an acute infection or were from someone with long COVID.

In summary, the Panther Fusion SARS/Flu/RSV assay demonstrated robust clinical performance in this large multi-center clinical study and represents a new FDA-cleared option for comprehensive, mid- to high-level throughput, multiplex respiratory testing for SARS-CoV-2, influenza A virus, influenza B virus, and RSV in nasopharyngeal swab specimens from symptomatic individuals.

## SUPPLEMENTAL MATERIAL

Supplemental file 1 jcm.00827-23-s0001.docx Tables S1 to S4 and Fig. S1

ASM does not own the copyrights to Supplemental Material that may be linked to, or accessed through, an article. The authors have granted ASM a non-exclusive, world-wide license to publish the Supplemental Material files. Please contact the corresponding author directly for reuse.
