# Supplementary material for: Multi-center clinical evaluation of the Panther Fusion SARS-CoV-2/Flu A/B/RSV assay in nasopharyngeal swab specimens from symptomatic individuals
Source: J Clin Microbiol. 2023 Oct 30;61(11):e00827-23. doi: 10.1128/jcm.00827-23 (PMC10662375; doi:10.1128/jcm.00827-23)
Supplement: Supplemental file 1 — Tables S1 to S4 and Fig. S1 [file jcm.00827-23-s0001.docx]

**Supplementary Material**

**Supplement Table 1. Listing of Specimens with Discordant Results for SARS-CoV-2**

|  |  |  |  | **SARS-CoV-2 CCA Results** | | | |  | | |  |
| --- | --- | --- | --- | --- | --- | --- | --- | --- | --- | --- | --- |
| **Outcome** | **Subject ID** | **Specimen Type** | **PF SARS/Flu/RSV Result (Ct)** | **cobas  (Ct1, Ct2)^1^** | **Aptima** | **PCR/BDS** | **Infected Status** | | **Discordant Testing Result^2^** | | |
| FN | 1930015 | Prospective Fresh | Neg | PrPos  (Neg, 37.58) | Pos | Pos | Infected | | ND | | |
|  | 1930020 | Prospective Fresh | Neg | Neg | Pos | Pos | Infected | | ND | | |
|  | 4680199 | Prospective Fresh | Neg | Neg | Pos | Pos | Infected | | ND | | |
|  | 4680441 | Prospective Frozen | Neg | Neg | Pos | Pos | Infected | | ND | | |
|  | 4682298 | Prospective Frozen | Neg | PrPos  (Neg, 36.58) | Pos | Neg | Infected | | ND | | |
|  | 4682607 | Prospective Frozen | Neg | PrPos  (Neg, 37.91) | Neg | Pos | Infected | | ND | | |
|  | 4802131 | Prospective Frozen | Neg | Neg | Pos | Pos | Infected | | Pos (33) | | |
|  | 4802164 | Prospective Frozen | Neg | PrPos  (Neg, 35.97) | Pos | Pos | Infected | | Pos (36.8) | | |
|  | 4802228 | Prospective Frozen | Neg | Pos (34.4) | Pos |  | Infected | | Pos (34.8) | | |
|  | 4802304 | Prospective Frozen | Neg | PrPos  (Neg, 37.91) | Pos | Pos | Infected | | Pos (38.2) | | |
|  | 4805041 | Prospective Frozen | Neg | Pos (35.36) | Pos |  | Infected | | Pos (37.1) | | |
|  | 4910022 | Prospective Fresh | Neg | QNS | Pos | Pos | Infected | | ND | | |
| FP | 1930013 | Prospective Fresh | Pos (36.6) | Neg | Pos | Neg | Not infected | | Pos (36.3) | | |
|  | 1930073 | Prospective Fresh | Pos (39.1) | PrPos  (Neg, 38.74) | Neg | Neg | Not infected | | Neg | | |
|  | 1930263 | Prospective Fresh | Pos (39.2) | Neg | Pos | Neg | Not infected | | ND | | |
|  | 1930265 | Prospective Fresh | Pos (39.1) | Neg | Neg |  | Not infected | | Neg | | |
|  | 1930376 | Prospective Fresh | Pos (40.3) | Neg | Pos | Neg | Not infected | | ND | | |
|  | 4680052 | Prospective Fresh | Pos (39.2) | Neg | Neg | NA | Not infected | | Neg | | |
|  | 4680068 | Prospective Fresh | Pos (39.3) | Neg | Neg | NA | Not infected | | ND | | |
|  | 4680417 | Prospective Frozen | Pos (39.2) | Neg | Neg | NA | Not infected | | ND | | |
|  | 4680432 | Prospective Frozen | Pos (32.4) | Neg | Neg | NA | Not infected | | ND | | |
|  | 4680464 | Prospective Frozen | Pos (39.3) | Neg | Neg | NA | Not infected | | ND | | |
|  | 4680494 | Prospective Frozen | Pos (38) | Neg | Pos | Neg | Not infected | | Pos (37.4) | | |
|  | 4680575 | Prospective Frozen | Pos (39.4) | Neg | Neg | NA | Not infected | | Neg | | |
|  | 4682237 | Prospective Frozen | Pos (36.3) | PrPos  (Neg, 34.78) | Neg | Neg | Not infected | | Pos (40.1) | | |
|  | 4682256 | Prospective Frozen | Pos (39.3) | Neg | Neg | NA | Not infected | | Pos (44.2) | | |
|  | 4682424 | Prospective Frozen | Pos (39.7) | Neg | Pos | Neg | Not infected | | ND | | |
|  | 4682525 | Prospective Frozen | Pos (39.1) | Neg | Neg | NA | Not infected | | ND | | |
|  | 4702623 | Prospective Frozen | Pos (38.8) | Neg | Pos | Neg | Not infected | | Pos (41.6) | | |
|  | 4802727 | Prospective Frozen | Pos (37.1) | PrPos  (Neg, 37.85) | Neg | Neg | Not infected | | ND | | |
|  | 4802937 | Prospective Frozen | Pos (37.8) | Neg | Neg | NA | Not infected | | Pos (37.9) | | |
|  | 4805324 | Prospective Frozen | Pos (35.9) | Neg | Pos | Neg | Not infected | | Pos (39.4) | | |
|  | 4910067 | Prospective Fresh | Pos (39.3) | Neg | Neg | NA | Not infected | | ND | | |
|  | 4910110 | Prospective Fresh | Pos (37.4) | Neg | Neg | NA | Not infected | | ND | | |
|  | 4910116 | Prospective Fresh | Pos (39.8) | Neg | Neg | NA | Not infected | | ND | | |
| \| BDS = bidirectional sequencing, CCA = composite comparator algorithm, FP = false positive, FN = false negative, NA = not applicable, ND = testing not done (no volume), Neg = negative, PrPos = presumptive positive, Pos = positive, QNS = quantity not sufficient for testing. Note: If available, Ct values are shown in parentheses for all specimens with Positive testing results. ^1^Ct1 corresponds to the SARS-CoV-2 specific ORF1ab target 1. Ct2 corresponds to the conserved pan-Sarbecovirus E gene region that is not specific to SARS-CoV-2.  ^2^Discordant testing was performed using EUA Xpert Xpress SARS-CoV-2/Flu/RSV assay, volume permitting. \| \| --- \| | | | | | | | | | |  |  |

**Supplement Table 2. Listing of Specimens with Discordant Results for Influenza A Virus**

| **Outcome** | **Subject ID** | **Specimen Type** | **PF SARS/Flu/RSV Result (Ct)** | **PF Flu A/B/RSV Result (Ct)** | **Discordant Testing Result^1^** | |
| --- | --- | --- | --- | --- | --- | --- |
| FN | 1930221 | Prospective Fresh | Neg | Pos (38.7) | ND | |
|  | 4804098 | Retrospective | Neg | Pos (37.2) | Pos (38) | |
|  | 4910158 | Prospective Fresh | Neg | Pos (35.4) | ND | |
| FP | 4680104 | Prospective Fresh | Pos (42.7) | Neg | ND | |
|  | 4680123 | Prospective Fresh | Pos (39.5) | Neg | ND | |
|  | 4680407 | Prospective Frozen | Pos (44.1) | Neg | ND | |
|  | 4802096 | Prospective Frozen | Pos (38.7) | Neg | Neg | |
|  | 4802738 | Prospective Frozen | Pos (43) | Neg | Neg | |
| FP = false positive, FN = false negative, ND = not done (no volume), Neg = negative, PF = Panther Fusion, Pos = positive. Note: If available, Ct values are shown in parentheses for all specimens with Positive testing results. ^1^Discordant testing was performed using EUA Xpert Xpress SARS-CoV-2/Flu/RSV assay, volume permitting. | | | | | |  |

**Supplement Table 3. Listing of Specimens with Discordant Results for Influenza B Virus**

| **Outcome** | **Subject ID** | **Specimen Type** | **PF SARS/Flu/RSV Result (Ct)** | **PF Flu A/B/RSV Result (Ct)** | **Discordant Testing Result^1^** | |
| --- | --- | --- | --- | --- | --- | --- |
| FN | 4804030 | Retrospective | Neg | Pos (39.5) | Pos (37.9) | |
| FP | 1930199 | Prospective Fresh | Pos (42.4) | Neg | Neg | |
|  | 4680065 | Prospective Fresh | Pos (42) | Neg | ND | |
|  | 4680405 | Prospective Frozen | Pos (40.1) | Neg | ND | |
|  | 4682749 | Prospective Frozen | Pos (42.2) | Neg | ND | |
| FP = false positive, FN = false negative, ND = not done (no volume), Neg = negative, PF = Panther Fusion, Pos = positive. Note: If available, Ct values are shown in parentheses for all specimens with Positive testing results. ^1^Discordant testing was performed using EUA Xpert Xpress SARS-CoV-2/Flu/RSV assay, volume permitting. | | | | | |  |

**Supplement Table 4. Listing of Specimens with Discordant Results for RSV**

| **Outcome** | **Subject ID** | **Specimen Type** | **PF SARS/Flu/RSV Result (Ct)** | **PF Flu A/B/RSV Result (Ct)** | **Discordant Testing Result^1^** | |
| --- | --- | --- | --- | --- | --- | --- |
| FN | 4680103 | Prospective Fresh | Neg | Pos (43.5) | ND | |
|  | 4680215 | Prospective Fresh | Neg | Pos (41.3) | ND | |
| FN = false negative, ND = not done (no volume), Neg = negative, PF = Panther Fusion, Pos = positive. Note: If available, Ct values are shown in parentheses for all specimens with Positive testing results. ^1^Discordant testing was performed using EUA Xpert Xpress SARS-CoV-2/Flu/RSV assay, volume permitting. | | | | | |  |

**Supplement Figure 1. Passing-Bablok Regression Ct Scatter Plots: Panther Fusion SARS/Flu/RSV vs Comparator, by Viral Target.**

| **SARS-CoV-2**  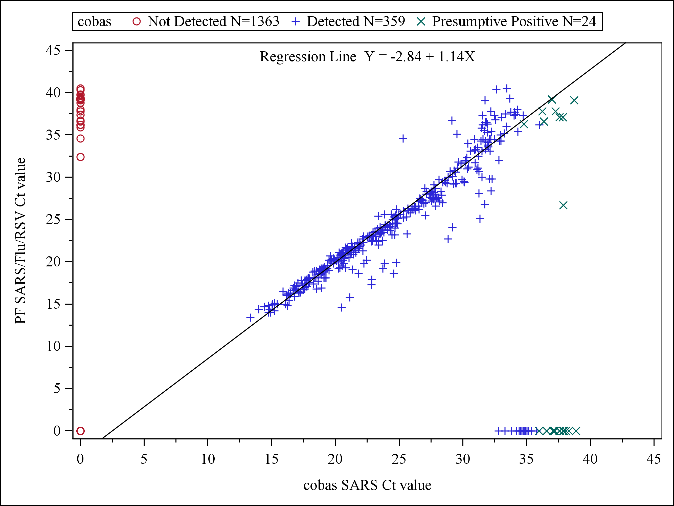 | **Influenza A Virus**  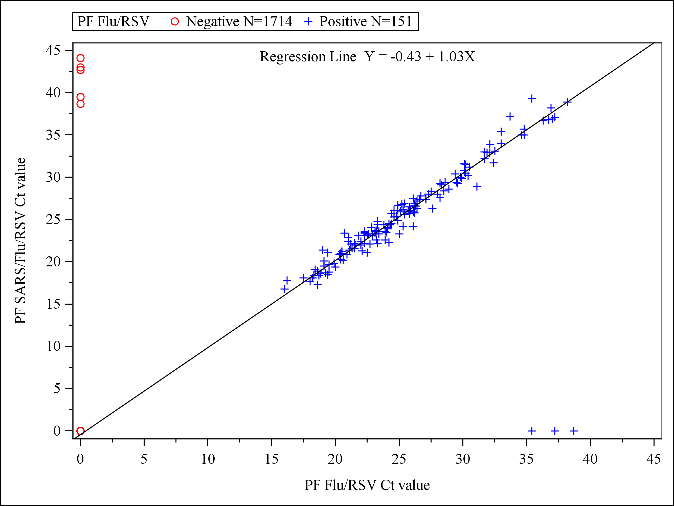 |
| --- | --- |
| **Influenza B Virus**  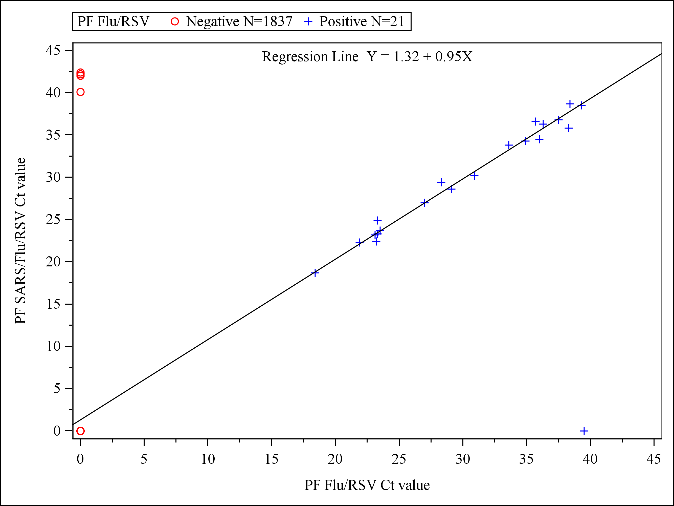 | **RSV**  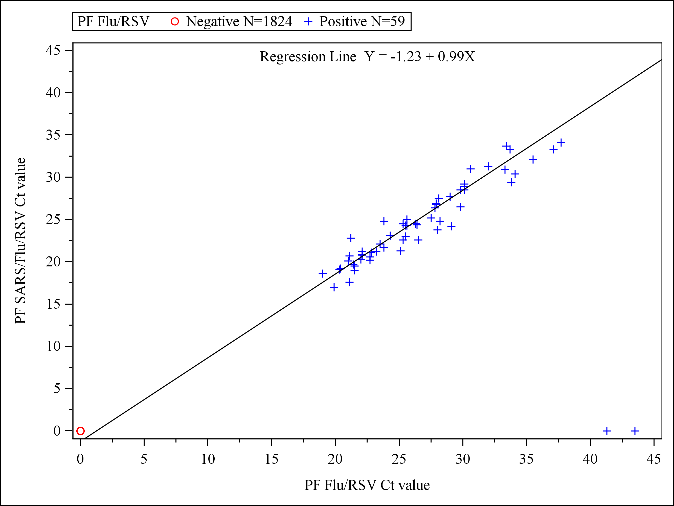 |

*SARS-CoV-2:* The scatter plot includes all evaluable prospective specimens. The regression line was calculated using all evaluable prospective specimens with positive SARS-CoV-2 results on the Panther Fusion SARS/Flu/RSV assay and the EUA cobas SARS-CoV-2 assay (target 1 – ORF1ab result). Comparison to the Aptima SARS-CoV-2 is not shown since it does not provide Ct values.

*Influenza A, Influenza B, and RSV:* The scatter plots include all evaluable prospective and retrospective specimens. The regression lines were calculated using all evaluable prospective and retrospective specimens with positive results on the Panther Fusion SARS/Flu/RSV assay and Panther Fusion Flu/A/B/RSV assay for influenza A virus, influenza B virus, and RSV, separately.
